# Investigating context-specific acoustic variation in the bleats of male domestic sheep

**DOI:** 10.1515/biol-2025-1368

**Published:** 2026-08-31

**Authors:** Apostolos Ntairis, Anastasia Frantzola, Ioannis Mavrikos, George P. Laliotis

**Affiliations:** Laboratory of Animal Breeding and Husbandry, Department of Animal Science, Agricultural University of Athens, 75 Iera Odos, GR11855, Athens, Greece

**Keywords:** rams, vocalizations, emotions, *Ovis aries*, isolation

## Abstract

Vocalizations provide an informative means of assessing the emotional states of animals, yet male vocal behaviour in domestic sheep remains unexplored. The study aimed to investigate whether rams’ bleats vary across five distinct contexts associated with morning and night isolation, exposure to familiar ewe-bell sounds, feed anticipation, and feed denial. A total of 428 high-quality bleats were used to extract 20 acoustic parameters, which were then analyzed using a multivariate acoustic approach. Principal Component Analysis identified five independent acoustic components reflecting source-parameters, spectral energy, formant frequencies, voice stability, and temporal characteristics. Context significantly influenced the acoustic structure of bleats, with spectral, formant, vocal stability, and temporal characteristics showing context-dependent variation, whereas fundamental frequency-related parameters remained unaffected. Feed anticipation elicited higher formant-related scores; feed denial produced reduced spectral energy, and social isolation contexts were characterized by increased vocal instability. Discriminant analyses revealed moderate classification accuracy, indicating overlapping acoustic patterns and supporting graded rather than discrete vocal signals. Morphometric effects were minimal, with wither height affecting formant structure. The findings provide the first detailed evidence that rams modulate multiple acoustic features across contexts, demonstrating that male sheep encode emotional states through shifts in vocal parameter variation.

## 1 Introduction

Vocal communication constitutes a fundamental component of mammalian behaviour, enabling individuals to interact with conspecifics and their environment and to further convey information about their internal state. Vocal signals arise from the coordinated action of the respiratory system, larynx, and supralaryngeal vocal tract, and their structure is shaped by the anatomical and physiological constraints of the vocal apparatus described by the source–filter theory [1], [2], [3]. The vibration of the vocal folds (source) generates a periodic excitation signal that defines the fundamental frequency (F0), while the configuration and dynamic shaping of the vocal tract (filter) selectively modify this signal by imposing resonance patterns. These resonances determine the formant frequencies and the spectral envelope, allowing variation in vocal-tract morphology or posture to alter the acoustic output independently of the underlying vibratory source. The involved anatomical and physiological mechanisms provide a biological framework through which emotional and motivational states can be expressed acoustically.

Across mammals, vocalizations can serve as reliable indicators of emotional states [3], which are commonly conceptualized along two primary dimensions, valence and arousal [4], 5]. Valence distinguishes between positive and negative experiences, whereas arousal reflects the intensity of the underlying motivational activation. Together, these dimensions shape the physiological processes underlying vocal production, leading many species to systematically modulate acoustic parameters across affective contexts. Mammalian species, however, differ in how they encode such affective information. Some species, including pigs, cattle, and several primates, produce relatively discrete call types associated with specific motivational states [6], [7], [8]. In contrast, small ruminants such as goats and sheep often exhibit vocal signals in which acoustic parameters vary continuously along emotional dimensions rather than forming sharply call types [3], 9], 10]. Changes in valence and arousal influence the respiratory system, laryngeal tension, and vocal tract configuration, thereby altering the acoustic structure of vocalizations through modifications in fundamental frequency, formant patterns, spectral energy distribution, and temporal characteristics.

Sheep have a comparatively narrow vocal repertoire compared with other species such as pigs and primates. Their vocalizations are discriminated in: (a) bleats of low-pitched or ‘rumble’ calls, which are typically produced by ewes in close contact with their lambs and thought to support maternal care and bonding, and (b) high-pitched bleats commonly associated with distress, particularly during separation or challenging situations [11], [12], [13]. Much of the research conducted on sheep vocal communication has focused on the ewe–lamb bond, where vocalizations play a central role in maternal recognition, offspring location, and the regulation of proximity and caregiving behaviour. Ewes produce individually distinctive low-frequency bleats that facilitate selective recognition of their lambs, while lambs emit higher-pitched bleats when separated or distressed [12], [13], [14]. Emotional modulation of these calls is well documented, with separation distress increasing fundamental frequency, perturbation measures, and number of bleats, whereas maternal contact reduces vocal intensity. Recent work has further shown that acoustic parameters vary across different physiological or management contexts, including lamb separation, social isolation of ewes, and ewes’ isolation during the dry period, with consistent alterations observed in frequency-related parameters and vocal instability [10]. Further, changes in ewes’ social group composition also influenced vocal signals. When familiar group members were removed and replaced with unfamiliar ones, ewes produced bleats with elevated fundamental frequency and amplitude, consistent with negative emotional valence and distress [15].

Although vocal communication in ewes and lambs has been well-studied, especially considering maternal, neonatal and ewes’ calls under different situations, far less is known about whether and to what extent males express emotional states through their vocalizations. Extending acoustic analyses to rams is therefore essential for broadening our understanding of vocal expression beyond the maternal domain and for clarifying how emotional processes shape vocal behaviour across sexes. The present study investigated whether rams’ bleats differ across different contexts associated with social isolation, exposure to familiar sounds, feed anticipation and feed denial. Our aim was to assess how the acoustic structure of rams’ bleats varies across these contexts and to determine whether changes in acoustic parameters form discrete context-specific patterns or instead reflect a varied emotional expression. Based on previous findings in mammals, we expected negatively valenced contexts, such as social isolation or feed frustration, to elicit more unstable or reduced spectral-related parameters, whereas positively valenced contexts were anticipated to produce more resonant or temporally distinct calls. By characterizing how source–, filter–, and temporal–related acoustic parameters shift across different conditions, the study expects to provide new insight into rams’ vocalizations and the extent to which emotional states are encoded through vocal cues in sheep.

## 2 Materials and methods

### 2.1 Study site, animals, and experimental set-up

All data were collected on a commercial dairy sheep farm in Voiotia, Greece (spring 2024), prior to the mating period. The farm maintained a breeding population of 120 ewes and 15 unrelated adult rams, all belonging to the Assaf breed according to the farmer’s records. Also, the composition of this male group was stable, having remained unchanged for at least three years prior to the study. Vocalizations were recorded from all 15 rams, which were housed together in a separate male group (bachelor group) (N 38°22′2.046″, E 23°7′10.83″), located approximately 600 m from the ewes’ enclosures (N 38°21′46.534″, E 23°6′58.388″). The rams were 1.5–5 years old (mean ± SE: 3.27 ± 0.33 years). Wither height, body and head length were measured one day before recordings.

Acoustic data were collected using a ZOOM SSH-6 microphone (20–20,000 Hz) connected to an H5 Handy Recorder, positioned centrally within the pen at 130 cm height and fitted with a windshield to minimize artefacts. A wired remote control enabled unobtrusive operation. All vocalizations were stored as uncompressed.WAV files (44.1 kHz, 16 bit). Recordings were conducted only under suitable weather conditions.

To avoid overlapping calls, each ram was visually and physically isolated in a barn pen; thus, each ram was placed alone in an individual pen so that no other animals could be seen or approached, and no physical contact with conspecifics was possible. Each ram was temporarily moved from the group pen to a separate enclosed isolation pen located within the same farm, approximately 30 m away from the males’ group housing area. Separation of the rams was carefully conducted by the farmer with whom the animals were habituated, to avoid additional handling stress and anxiety. The animals were familiar with the pen, since it is routinely used for rams’ isolation during farm management (e.g., inspections, vaccinations etc.) and, thus, no novelty effects were expected. Vocalizations were recorded under five contexts: a) morning isolation (9:00 h; hereafter designated as Context A); b) isolation while hearing familiar sounds of ewe-bell produced by the farmer at 20–30 m distance, without visual contact (hereafter designated as Context B); c) evening isolation (20:00 h; hereafter designated as Context C); d) isolation with feed anticipation (alfalfa hay; hereafter designated as Context D); and e) isolation with feed denial (hereafter designated as Context E). Experimentations were conducted between 08:30–14:00 h (Contexts A, B, D, E) and between 20:00–21:00 h (Context C). Each ram was isolated for 3 min per context and then returned to the male group. All animals were tested for each context on the same day following a predefined randomized order generated prior to the start of the experiment and maintained constant across all examined contexts, to avoid order effects and ensure that isolation was not systematically linked to any individual’s position in the group.

**Ethical approval:** The research related to animal use has been complied with all the relevant national regulations and institutional policies for the care and use of animals, and has been approved by the Bioethical Committee of the Agricultural University of Athens (approval code 5/14-1-2021).

### 2.2 Contextual classification of recordings based on emotional valence

Recording contexts were assigned to positive or negative based on their inferred emotional valence [3]. Valence attribution for isolation, auditory cues, feed anticipation, and feed denial was based on established theoretical frameworks on the functional role of emotions [3], 5], 16], 17] and empirical knowledge of livestock behavioural responses [3], 10], 17], 18]. Negative valence corresponds to activation of the unpleasant–avoidance motivational system, which drives withdrawal from stimuli that sheep naturally avoid. Positive valence reflects activation of the pleasant–appetitive system, which promotes approach toward stimuli that confer adaptive benefits [5], 16], 17].

Social isolation is inherently aversive for sheep; however, valence attribution in this study was based on the motivational properties of the stimuli introduced during isolation. On this basis, contexts involving physical or social isolation and feed denial were classified as negative, whereas feed anticipation and exposure to familiar related sounds were classified as positive. Feed anticipation was considered positively valanced because feeding reliably elicits approach behaviour and contributes to fitness. It constitutes an appetitive state associated with the expectation of reward, which is ultimately given to the animal. Therefore, it triggers a positive expectation and reward mechanism, activating the pleasant appetitive system. Familiar auditory cues associated with females (ewes) were also deemed positive due to their relevance for reproductive motivation, since they can trigger reproductive motivation, which represents a highly salient positive appetitive stimulus for adult rams, especially when mating season is expected. Conversely, social isolation was considered negatively valenced given the species’ strong gregariousness and the potential fitness risks of separation. Feed denial was similarly classified as negative due to its association with frustration, reduced intake, and potential fitness costs [5], 9], 17].

### 2.3 Extraction of acoustic parameters

Acoustic analyses were performed using Praat (v.6.4.63) [19]. Spectrograms were generated using an FFT approach (window length 0.03 s, 1000-time steps, 250 frequency steps, Gaussian window, 50 dB dynamic range). Only bleats with high quality and minimal background interference were retained, resulting in 428 high-pitched bleats (96 % of all recorded vocalizations). 20 acoustic parameters related to source, filter, and intensity characteristics (Table 1) were extracted using Praat commands and custom scripts [10], 15], 20], 21].

**Table 1:** Descriptions and abbreviations of all acoustic parameters extracted from each vocalization.

Acoustic parameter	Definition
Dur (s)	Mean duration of the call
Q25 (Hz)	Frequency value at the upper limit of the first quartile of energy
Q50 (Hz)	Frequency value at the upper limit of the second quartile of energy
Q75 (Hz)	Frequency value at the upper limit of the third quartile of energy
fpeak (Hz)	Frequency of peak amplitude
F0TimeMax (s)	Time point at which the fundamental frequency (F0) reaches its maximum value.
F0mean (Hz)	Mean F0 frequency across the call
F0AbsSlope (Hz/s)	F0 mean absolute slope
F0Range (Hz)	Difference between the maximum F0 and the minimum F0 frequency
F0var (Hz/s)	Average fundamental frequency variation per unit time
FMExtent (Hz)	Mean peak-to-peak variation in each F0 modulation
Jitter%	Mean absolute difference between frequencies of consecutive F0 periods divided by the mean frequency of F0mean
Shimmer%	Mean absolute difference between the amplitudes of consecutive F0 periods divided by the mean amplitude of F0
F1mean (Hz)	Mean frequency value of the first formant
F2mean (Hz)	Mean frequency value of the second formant
F3mean (Hz)	Mean frequency value of the third formant
F4mean (Hz)	Mean frequency value of the fourth formant
AmpVar (dB/s)	Mean intensity variation per second
AMRate (s^−1^)	Number of complete cycles of amplitude modulation per second
VTLmax (cm)	Estimated maximum length of vocal tract

Source-related parameters were derived from the F0 contour generated via the “To Pitch (cc)” command (time step 0.01 s; voice threshold 0.2; silence threshold 0.05; F0 range 65–550 Hz). From this contour, F0mean, F0Range, Q25, Q50, Q75, F0TimeMax, FMRate and fpeak were calculated. The F0var was calculates as described in [22]. Jitter % and Shimmer % were obtained as measures of vocal perturbation using the Praat commands “Jitter (local)” and “Shimmer (local), respectively. Filter-related parameters were extracted using the “To Formant (cc)” (time step 0.01 s; maximum formants 4; maximum formant 5,000 Hz; window length 0.05 s) and the mean values of F1–F4 were computed. Spectrograms were visually inspected to ensure accurate formants’ and F0 tracking. Intensity-related features were derived using “To Intensity (cc)” command, including AmpVar and AMRate. The duration of each bleat (Dur) was also extracted. Maximum vocal tract length (VTLmax) was estimated using the following equation: VTLmax = c/2·ΔFmin and the regression approach described previously [22]. VTLmax was analyzed separately to account for inter-individual differences and was not included in the main acoustic dataset. Each bleat was treated as an independent statistical unit.

### 2.4 Statistical analyses

All statistical analyses were conducted using SPSS v.26 (IBM Corp, 2019). In each tested context, only individuals that contributed at least three vocal calls per vocal bout were retained. This approach optimized data retention while limiting the loss of individuals with sparse observations [21]. The data were checked for normal distribution (Q–Q plots) and log-transformed if necessary (F0mean, F0Range, F0Var, FMExtent). Pairwise comparisons were conducted with Bonferroni-adjusted significance levels to correct for repeated measures (*p* < 0.05). Each bleat (call) was treated as separate unit in the statistical analysis.

#### 2.4.1 Principal component analysis (PCA)

To address redundancy arising from strong inter-correlations among acoustic parameters, all variables were subjected to a PCA. First, the Keiser-Mayer-Olkin measure of sampling adequacy was used to assess the suitability of the acoustic parameters’ dataset for multivariate reduction. (PCA; overall KMO score: 0.81). Since the overall KMO score was higher than 0.5 [23], a PCA was further performed on the extracted acoustic parameters using a correlation matrix and the Varimax rotation method. Principal components with eigenvalues greater than 1 were retained for subsequent statistical analyses.

#### 2.4.2 Differences in acoustic parameters across contexts

To assess whether the overall acoustic structure of the bleats differed across behavioural contexts, we conducted a multivariate analysis of covariance (MANCOVA) using the five principal component (PC) scores as dependent variables. MANCOVA provides a global test of multivariate separation and evaluates whether contexts differ in their combined acoustic profiles rather than in individual acoustic dimensions alone. Context was included as a fixed factor, and wither height was entered as a covariate to account for potential effects of body size on the multivariate acoustic space. Pillai’s Trace test was used to assess the overall multivariate effect of context on the combined acoustic variables (PC1–PC5). Since MANCOVA does not account for multiple bleats per animal across contexts, we subsequently analyzed each PC using linear mixed-effects models. Linear Mixed Models (LMMs) fitted with Restricted Maximum Likelihood were used to investigate (a) how morphometric traits (wither height, body length, head length) may affect the acoustic parameters and (b) how context influences source, filter, and intensity features, as well as VTLmax. In both modelling frameworks, principal components with eigenvalues greater than 1 were used as response variables, and ram identity (ID) was included as a random effect to control for repeated observations within individuals. The morphometric measurements were entered as fixed effects in the first model. In the second model, the context was set as a fixed effect, as well as wither height as a covariate to account for the different body sizes of the animals.

#### 2.4.3 Discriminant function analysis (DFA)

To evaluate the degree to which the examined contexts exhibited distinct or overlapping acoustic signatures, we conducted a Discriminant Function Analysis (DFA) using the five principal component (PC) scores as predictors and context as the grouping variable. We first derived a single acoustic profile for each individual within each context by averaging their PC scores, thereby ensuring that each case represented an independent ID × context combination. These PC values were then entered into the DFA to derive canonical discriminant functions, group centroids, and structure coefficients. The analysis assessed the degree of multivariate separation among contexts and identified which acoustic dimensions contributed most strongly to classification. Classification performance and the degree of acoustic similarity among contexts were assessed using cross-validated (leave-one-out) classification accuracy.

#### 2.4.4 Proximity analyses

Following the DFA, discriminant scores for Function 1 and Function 2 were aggregated by context to obtain the mean discriminant score for each function, representing the DFA centroid of each context. Euclidean distances between these centroids were subsequently calculated to quantify multivariate separation among contexts. The resulting distance matrix reflects the relative acoustic similarity or distinctiveness of each context pair in the discriminant space. Two complementary multivariate proximity analyses were conducted on the PCs’ mean vectors (PC1_mean_-PC5_mean_) using (i) a Euclidean distance dissimilarity analysis to measure pairwise multivariate separation among PCs and (ii) a cosine similarity analysis to quantify the similarity among principal component vectors and identify the degree to which principal components exhibited aligned or opposing patterns. These analyses provided an additional assessment of structural relationships among the acoustic components beyond their discriminant contributions.

## 3 Results

### 3.1 Principal component analysis

Descriptive statistics of the rams’ acoustic parameters across the five experimental contexts are presented in Supplementary Table 1. The principal component analysis (PCA) performed on the vocal parameters identified five principal components (PC1–PC5) with eigenvalues greater than 1 (KMO = 0.812; *p* < 0.001). These components accounted for 74.52 % of the variance in the dataset. The corresponding variable loadings for each component are shown in Table 2. Variable loadings indicated that the first principal component (PC1) was highly correlated (*|r*_*s*_ *|* > 0.50) with fundamental frequency characteristics, including the F0 mean, F0 range, F0 variation, and modulation parameters, reflecting, therefore, source-related dynamics. Calls with higher PC1 scores were indexed by higher F0 mean values, greater pitch variability and wider F0 range. The second principal component (PC2) represented the distribution of spectral energy across the call, with loadings being higher on the lower, median, and upper frequency quartiles (Q25, Q50 and Q75, respectively) and the peak frequency of the spectrum (fpeak). Therefore, calls with higher PC2 values corresponded to calls with higher spectral energy. PC3 was characterized by high loadings on the mean values of the first four formant frequencies (F1mean – F4mean), corresponding to vocal-tract resonance structure. The fourth component (PC4) included voice-quality characteristics (F0 disturbance/perturbation), with Jitter% and Shimmer% contributing most strongly. The fifth principal component (PC5) was highly corelated with call duration (Dur), the timing of maximum F0 (F0TimeMax), and amplitude-modulation rate (AMRate), thus representing the temporal dynamics of the vocalizations. Higher PC5 scores corresponded to longer call durations, later occurrence of maximum value of fundamental frequency (F0) within the call, and faster amplitude-modulation rates.

**Table 2:** Principal component analysis results for the rams’ acoustic parameters (bolded values denote the strongest factor loadings; |*r*_*s*_| > 0.50).

Acoustic parameter	Principal component				
	PC1	PC2	PC3	PC4	PC5
Dur	0.002	−0.036	0.001	0.259	**0.795**
Q50	−0.179	**0.934**	−0.128	−0.021	−0.077
Q25	−0.100	**0.818**	−0.330	−0.032	−0.057
Q75	−0.088	**0.841**	0.137	−0.022	−0.068
fpeak	0.028	**0.682**	−0.246	−0.122	0.075
F0TimeMax	0.237	0.013	0.074	−0.100	**0.717**
F0mean	**0.839**	−0.037	−0.061	−0.111	0.182
F0AbSlope	**0.879**	−0.175	0.044	0.187	−0.026
F0Range	**0.833**	−0.043	0.071	0.147	0.214
F0var	**0.884**	−0.054	−0.011	0.354	0.042
FMExtent	**0.888**	−0.117	0.073	0.337	0.003
F1mean	−0.058	0.283	**0.804**	−0.105	0.044
F2mean	0.033	−0.278	**0.794**	−0.159	−0.054
F3mean	0.051	−0.284	**0.866**	0.094	0.069
F4mean	0.019	−0.239	**0.815**	0.189	0.066
Jitter%	0.283	−0.069	0.038	**0.848**	0.022
Shimmer%	0.440	−0.037	−0.043	**0.636**	0.168
AmpVar	**0.557**	−0.017	−0.064	0.426	0.200
AMRate	0.268	−0.206	0.061	0.485	**0.508**

### 3.2 Differences between contexts

A multivariate analysis of covariance (MANCOVA) revealed a significant overall effect of contexts on the multivariate acoustic components of the bleats (Pillai’s Trace = 0.364, *F* = 7.91, *p* < 0.001). Height also had a significant multivariate effect (Pillai’s Trace = 0.097, *F* = 8.44, *p* < 0.001), indicating that body size influenced the acoustic parameters simultaneously.

Linear mixed-effects models (LMMs) were followed up to identify which specific morphometric characteristics and acoustic components contributed to these multivariate differences. Body length did not affect the extracted PCs (PC1–PC5: *p* > 0.05; *F*_PC1_ = 3.41; *F*_PC2_ = 0.39; *F*_PC3_ = 0.34; *F*_PC4_ = 0.73; *F*_PC5_ = 1.01). The same was noticed for head length (PC1–PC5: *p* > 0.05; *F*_PC1_ = 1.95; *F*_PC2_ = 0.82; F_PC3_ = 0.96; *F*_PC4_ = 0.25; *F*_PC5_ = 1.21). The wither height was found to affect only PC3 (*F* = 9.25; *p* = 0.02). The VTLmax did not significantly differentiate among the contexts (*F* = 0.61, *p* > 0.05). Since VTLmax did not significantly differ between contexts, it was not included as a control factor in the LMMs that were conducted for the acoustic parameters.

Table 3 presents the effects of different contexts on the acoustic components (PC1–PC5) derived from the extracted acoustic parameters. Specifically, PC1 did not differ significantly (*F*_PC1_ = 0.92, *p* > 0.05) among contexts (Context A: 0.44 ± 0.20; Context B: −0.18 ± 0.17; Context C: 0.01 ± 0.18; Context D: −0.01 ± 0.15; Context E: 0.07 ± 0.16). In contrast, PC2 was affected by the context (*F*_PC2_ = 19.39, *p* = 0.000). Specifically, the calls in Context E exhibited significant lower (*p* = 0.000) PC2 values (−0.92 ± 0.23) than those of the other contexts (Context A: 0.03 ± 0.25; Context B: −0.28 ± 0.24; Context C: 0.11 ± 0.24; Context D: −0.21 ± 0.22). However, the PC2 values between Context A and Context D did not differ from one another. Also, PC3 values varied across contexts (*F*_PC3_ = 14.01, *p* = 0.000), with Context D showing significantly (*p* = 0.000) higher scores (0.66 ± 0.19) than those of Context B (−0.19 ± 0.21) and Context C (−0.003 ± 0.21). No significant differences of PC3 scores were noted among the Contexts A (0.27 ± 0.22), C (−0.003 ± 0.21), and E (0.30 ± 0.19). The PC4 scores were affected by the context (*F*_PC4_ = 7.47; *p* = 0.000). Specifically, the mean values of PC4 scores were higher in Context A (0.50 ± 0.22) and Context C (0.43 ± 0.21) compared to those in the Context B (−0.01 ± 0.20; *p* < 0.05), Context D (−0.09 ± 0.19; *p* < 0.01)), and Context E (−0.15 ± 0.19; *p* < 0.01). Finally, the PC5 scores differed among contexts (*F*_PC5_ = 4.62; *p* = 0.001). The lowest values were observed in Context C (−0.39 ± 0.17), which were significantly (*p* < 0.01) different than those observed in Context D (0.11 ± 0.15) and Context E (0.12 ± 0.16). The respective mean value in the Context A (−0.26 ± 0.19) did not differ significantly from that of Context D or Context E. The respective value in Context B (−0.14 ± 0.17) also did not differ significantly from any other context.

**Table 3:** Effect of context (A–E) on principal component scores (mean ± SE). Different superscripts within a column indicate significant differences (*p* < 0.05).

Context	PC1	PC2	PC3	PC4	PC5
A	0.44 ± 0.20	0.03 ± 0.25^a^	0.27 ± 0.22^a^	0.50 ± 0.22^a^	−0.26 ± 0.19^a,b^
B	−0.18 ± 0.17	−0.28 ± 0.24^a^	−0.19 ± 0.21^b^	−0.01 ± 0.20^b^	−0.14 ± 0.17^a,b^
C	0.01 ± 0.18	0.11 ± 0.24^a^	−0.003 ± 0.21^a,b^	0.43 ± 0.21^a,^	−0.39 ± 0.17^a^
D	−0.01 ± 0.15	−0.21 ± 0.22^a^	0.66 ± 0.19^a,c^	−0.09 ± 0.19^b^	0.11 ± 0.15^b^
E	0.07 ± 0.16	−0.92 ± 0.23^b^	0.30 ± 0.19^a,d^	−0.15 ± 0.19^b^	0.12 ± 0.16^b^

### 3.3 Context discrimination and acoustic parameters proximity across contexts

**Figure 1:**
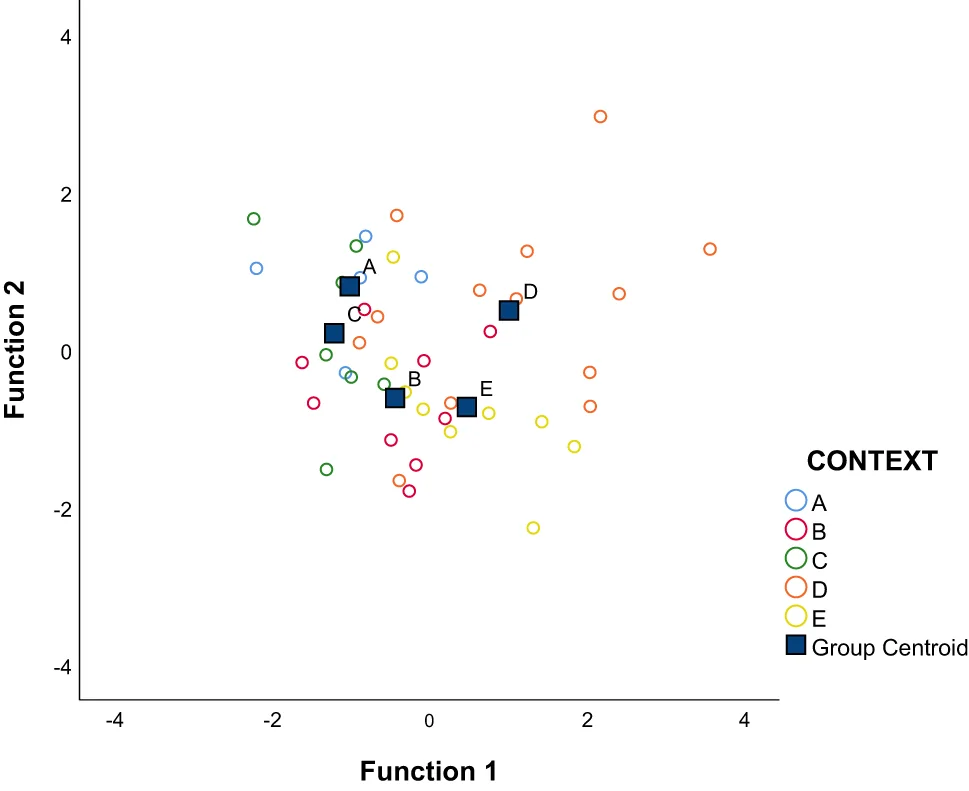
Canonical discriminant analysis (DFA) plot showing the distribution of calls across the first two canonical functions. Points represent individual PC profiles. Group centroids (blue squares) highlight the relative positioning of contexts in discriminant space, with greater distances indicating stronger acoustic differentiation. Axes correspond to the first two discriminant functions, which together explain 92.9 % of the total discriminating variance.

The discriminant function analysis (DFA) extracted four canonical functions, of which the first two accounted for most of the explained variance (Supplementary Table 2). Specifically, Function 1 had an eigenvalue of 0.851 and explained 63.6 % of the discriminating power (correlation: *r*_*s*_ = 0.68), while Function 2 had an eigenvalue of 0.392 and explained an additional 29.3 % (correlation: *r*_*s*_ = 0.53). Together, these two functions captured 92.9 % of the total multivariate discrimination among contexts. The overall test of all four functions was significant (Wilks’ λ = 0.354, χ² = 38.47, *p* = 0.008). The structure matrix (Table 4) indicated that Function 1 was primarily associated with PC3 (*r*_*s*_ = 0.67) and PC5 (*r*_*s*_ = 0.33) scores. These components represent variation in formant-related spectral structure (PC3) and temporal characteristics (PC5), revealing that formant frequences and call duration contributed most strongly to the separation of contexts along this axis. Function 2 was related to PC3 (*r*_*s*_ = 0.65) and PC4 (*r*_*s*_ = 0.39) scores, indicating that formant structure together with vocal stability (PC4) further differentiated contexts. Classification performance was modest (Figure 1), since the analysis (DFA) correctly classified 51.2 % of original cases and 32.6 % under leave-one-out cross-validation. Misclassifications were most common among intermediate contexts, whereas Context D showed the highest discrimination in both original (53.8 %) and cross-validated (46.2 %) classifications. DFA centroids’ distances (Supplementary Table 4; Figure 1) revealed differences in the degree of acoustic separation among contexts. The smallest distances were observed between Context A and Context C (0.627) and between Context B and Context E (0.918), showing that these contexts were acoustic similar and occupied adjacent regions of the discriminant space. In contrast, the largest distances occurred between Context C and Context D (2.237) and between Context A and Context E (2.134), demonstrating strong acoustic differentiation. Intermediate distances were noted between Context A and Context D (2.045) as well as between Context B and Context C (1.127), reflecting moderate separation.

**Table 4:** Structure matrix of correlations between the extracted principal component scores (PC1–PC5) and the discriminant functions. Highest absolute loadings (bolded) indicate the primary acoustic dimensions contributing to the two first discriminant functions.

PCs	Functions			
	1	2	3	4
PC3	**0.67**	**0.65**	0.10	−0.22
PC4	−0.28	**0.39**	0.66	0.52
PC1	0.03	−0.03	0.57	−0.47
PC2	−0.36	0.32	−0.52	−0.21
PC5	**0.33**	0.01	0.20	0.72

Further, the Euclidean distances between the PCA-derived centroids were used to summarize the relative proximity of contexts within the multivariate acoustic space of all examined contexts, providing a dissimilarity-based approach on PC components across contexts (Table 5). The smallest Euclidean distance was observed between PC4 and PC5 (4.482), confirming their close proximity, whereas the largest distance occurred between PC2 and PC3 (8.367), indicating substantial divergence between these components. Complementary, cosine similarity analysis (Table 5) revealed clear patterns of multivariate alignment among the principal component (PC) variables. Moderate positive cosine values were observed between PC4 and PC5 (0.450) and between PC1 and PC4 (0.271), indicating that these acoustic dimensions shared partially aligned directional structures in multivariate space. In contrast, negative cosine values (e.g., PC2 vs. PC3 = −0.278) reflected opposing vector orientations, suggesting that these components captured distinct acoustic patterns. Together, the cosine similarity and Euclidean dissimilarity matrices demonstrate a consistent pattern in which PC4 and PC5 form a closely related acoustic pair, while PC2 and PC3 represent the most structurally distinct dimensions.

**Table 5:** Proximity matrixes based on Euclidean distances (left) and cosine similarities (right) among the mean values of five principal component variables. Higher positive cosine values indicating greater similarity in direction and negative values indicating opposing orientations. Larger Euclidean distances indicate greater differences between PCs, whereas smaller distances reflect closer similarity in their underlying patterns.

PCs	Euclidean distance					Cosine of vectors				
	PC1	PC2	PC3	PC4	PC5	PC1	PC2	PC3	PC4	PC5
PC1	0	5.793	6.922	5.323	5.098	1	0.209	−0.019	0.271	0.179
PC2	5.793	0	8.367	6.647	6.477	0.209	1	−0.278	0.051	−0.067
PC3	6.922	8.367	0	7.464	6.397	−0.019	−0.278	1	−0.085	0.080
PC4	5.323	6.647	7.464	0	4.482	0.271	0.051	−0.085	1	0.450
PC5	5.098	6.477	6.397	4.482	0	0.179	−0.067	0.080	0.450	1

## 4 Discussion

The present study examined whether rams’ bleats vary across five contexts, related to negative valenced situations involving different temporal social isolation and feed denial (Contexts A, C, E), as well as with positively valenced conditions, including exposure to familiar related sounds and feed anticipation (Contexts B and D). By combining multivariate analyses, we characterized the multidimensional structure of the vocal signals and evaluated the extent to which acoustic components contribute to context differentiation. The results revealed context-related variation, suggesting that rams’ bleats vary across contexts rather than forming distinct call types.

Five acoustic components were revealed, reflecting source-related (F0) dynamics (PC1), spectral energy distribution (PC2), formant-related resonance structure (PC3), voice stability (PC4), and temporal characteristics (PC5). These dimensions are consistent with previous acoustic studies in mammals, where F0-related parameters, formant structure, and temporal features commonly emerge as independent axes of calls variation [2], 3], 10].

Context significantly influenced the acoustic structure of the bleats and, further, several PCs varied across contexts. The effect of context was evident for most components except PC1, indicating that fundamental frequency characteristics were relatively stable across contexts. This aligns with findings showing that the fundamental frequency (F0) is often more strongly influenced by arousal or individual identity than by specific states [9]. In contrast, the remaining acoustical components (PC2, PC3, PC4, and PC5) showed significant differences among contexts, suggesting that spectral energy distribution, formant structure, voice stability, and temporal characteristics are more sensitive to situational factors. Feed anticipation (Context D), a positively valenced situation, produced higher PC3 scores, suggesting changes in vocal tract configuration or resonance patterns. Conversely, feed denial (Context E), a negatively valenced condition, elicited lower PC2 values, indicating reduced spectral energy. Social isolation contexts (A and C), also negatively valenced, were characterized by higher PC4 values, reflecting increased vocal instability or perturbation. Similar context-dependent acoustic changes have been reported in sheep. Papadaki et al. [10] showed that social isolation, whether lamb separation or social isolation of ewes during the dry period, shifted changes in frequency-related parameters and in vocal perturbation measures (jitter, shimmer), with more pronounced disturbance under dry season. Recently, it has been reported that alterations in group composition influenced fundamental frequency and amplitude parameters, consistent with a negative emotional valence state [15]. Further, lamb calls have been reported to become higher in frequency, less tonal and shorter with increased negative arousal [24]. Such differentiation has been also reported in many other mammalian species including deer, goats, cattle, and pigs, where calls encode motivational or affective states through modulation of source- and filter-related parameters. Specifically, in red and fallow deer, both formant frequencies and temporal characteristics varied with motivational shifts [22], 25]. In cattle and pigs, it was reported that vocal responses to positive (e.g., feeding, social contact) and negative (e.g., isolation, frustration, feed restriction) contexts involved adjustments in fundamental frequency formants, and temporal structure [6], 8]. Further, goats’ calls vary with emotional arousal and valence, particularly in spectral and temporal parameters [9]. The present results therefore align with a well-established cross-species pattern, in which vocal signals reflect internal states and animals modulate their acoustic parameters in response to social and emotional challenges (e.g. changes in the social environment, feeding motivation).

Morphometric effects were limited, with only wither height influencing formant frequencies (PC3). This is consistent with previous studies where an established relationship between body size and formant frequencies in mammals has been reported [22], 26]. The absence of effects of body length, head length, and VTLmax may suggest that, within the examined population, anatomical variation was insufficient to produce systematic acoustic differences across contexts. This is consistent with the perception that, while formants can encode size information, the strength of this relationship depends on the degree of morphological variability within the sample [27]. Further, in intensive livestock systems, breeder selection for specific conformation traits that are related to reproduction performance, particularly in rams’ breeding group, may reduce natural variation in body size and vocal-tract-related morphology. Such selective pressures could potentially constrain the range of formant expression and weaken size–formant correlations, thereby limiting the extent to which anatomical differences are expressed acoustically in our dataset. Previous craniometric studies in sheep indicate that basal skull length corresponds to a static vocal-tract length, typically ranging from 17.16 to 24.6 cm in Romanov rams and Barbados Black Belly sheep, respectively [28], 29]. These anatomical estimates are lower than the VTL maximum values derived from the present study, suggesting that dynamic adjustments of the tract may occur during vocalization. Evidence from other mammals, including adult goats, a closely related species, shows that vocal-tract elongation can occur through changes in laryngeal position or pharyngeal configuration under different contexts [30]. However, the extent to which sheep employ similar mechanisms during bleating requires further investigation, as to the best of our knowledge, direct anatomic evidence of laryngeal retraction (i.e., video validation of dynamic vocal tract adjustments) is currently lacking.

The DFA further supported the role of formant-related and temporal features in context differentiation. PC3 and PC5 contributed most strongly to the first canonical function, while PC3 and PC4 loaded most strongly on the second. However, classification accuracy was modest, particularly under cross-validation, indicating substantial overlap among contexts. Previous studies reported similar modest discrimination patterns in sheep [10], 20], 31], but higher accuracy in other species including cattle [32], pigs [33], 34], and horses [35]. These cross-species differences likely reflect variation in vocal production. Sheep seem to produce graded vocal signals in which acoustic parameters shift continuously with changes in internal state rather than emitting discrete, context-specific call types compared to other species. Goats, despite being closely related to sheep, show a more diverse call repertoire and a stronger emotional modulation of fundamental frequency, amplitude, and formant pattern, which may increase vocal distinctiveness [9]. Species such as cattle, pigs, primates, and red deer also typically exhibit more discrete call types [32], [33], [34], [35], [36] and, in some cases (e.g. red deer), pronounced laryngeal retraction [22], producing clearer acoustic boundaries and therefore higher classification accuracy.

The DFA centroid distances provided insights into how rams modulate their vocal responses across the different contexts tested, confirming that rams’ vocalizations vary systematically with emotional and motivational conditions, consistent with established findings in sheep and other mammals [3], 6], 10]. Contexts with the smallest separations (e.g., Contexts A–C and B–E) clustered closely in the discriminant space, indicating acoustic similarity and suggesting overlapping affective states, a pattern aligned with the notion that vocal signals often encode graded emotional information rather than discrete categories [5], 9]. The observed close proximity of negative contexts related to morning and night isolation (Contexts A, C) in the discriminant space likely reflects comparable levels of arousal and motivational activation. Probably, arousal is encoded more strongly than valence, and when valence differences are moderate, the resulting vocal cues remain acoustically similar, producing small centroid separations. The time of isolation (morning or afternoon) did not appear to differentiate the calls, suggesting that during the examined period, circadian influences were unlikely to affect vocal parameters. However, the latter needs further investigation. Context B (exposure to familiar ewe-bell sounds) and Context E (feed denial), despite representing opposite emotional valence, showed unexpectedly close DFA centroid distances, indicating partially overlapping acoustic characteristics. This proximity potentially reflects similarities in arousal-related vocal adjustments rather than in valence. Similar proximity between positive and negative contexts has been documented in other species when arousal levels converge, supporting that arousal-related vocal adjustments can overshadow valence differences [3], 34]. Also, the rams in Context B and Context E produced bleats that did not differ in source-related parameters (PC1), perturbation characteristics (PC4), and temporal parameters (PC5), resulting in convergence within the multivariate acoustic space. In contrast, the largest distances (e.g., Contexts C–D and A–E) reflected acoustic differentiation, consistent with more distinct motivational conditions. These contexts engage different affective systems or distress (Contexts A and C: isolation) versus reward seeking (Context D), resulting in highly distinct acoustic patterns in the discriminant space. This is in accordance with previous studies where different contexts lead to acoustic divergence in sheep, goats, pigs, cattle, and deer calls [9], 15], 32], 37]. These geometric patterns aligned with the DFA accuracy results, where closely spaced contexts showed higher confusion and widely separated contexts were classified more accurately, confirming that the discriminant space captures meaningful context-dependent structure in the calls. Together, these findings indicate that the multivariate acoustic space captures meaningful variation in context-dependent vocal expression, supporting the interpretation that sheep calls encode both graded and discrete components of emotional signaling.

The proximity analyses provided additional insight into the structure of the acoustic components. The close association between voice instability (PC4) and temporal characteristics (PC5) suggests that perturbation measures and time-related parameters co-vary across contexts. Such a pattern has also been observed in goats and deer under different emotional contexts [3], 24], 37]. Conversely, the strong divergence between spectral energy (PC2) and formant structure-related characteristics (PC3) reflects the functional independence of these acoustic dimensions, which is consistent with previous studies across mammalian species [2], 7], 25].

Overall, our findings contribute to a broader understanding of vocal communication in domestic sheep and how acoustic features vary across contexts and particularly in spectral, formant-related, and temporal dimensions by providing new evidence from male vocalizations, for which empirical data have been limited. However, the moderate classification suggests that these acoustic differences may reflect graded changes rather than discrete context-specific call types. Further research incorporating complementary anatomical and contextual data will be essential to clarify the mechanisms underlying acoustic variation and to develop a more comprehensive understanding of vocal communication in small ruminants.

## Supplementary Material

Supplementary Material

## References

[1] Fant G (1960). Acoustic theory of speech production.

[2] Taylor AM, Reby D (2010). The contribution of source–filter theory to mammal vocal communication research. J Zool.

[3] Briefer EF (2012). Vocal expression of emotions in mammals: mechanisms of production and evidence. J Zool.

[4] Paul ES, Harding EJ, Mendl M (2005). Measuring emotional processes in animals: the utility of a cognitive approach. Neurosci Biobehav Rev.

[5] Mendl M, Burman OHP, Paul ES (2010). An integrative and functional framework for the study of animal emotion and mood. Proc R Soc B.

[6] Manteuffel G, Puppe B, Schön PC (2004). Vocalization of farm animals as a measure of welfare. Appl Anim Behav Sci.

[7] Fischer J, Noser R, Hammerschmidt K (2013). Bioacoustic field research: a primer to acoustic analyses and playback experiments with primates. Am J Primatol.

[8] Friel M, Kunc HP, Griffin K, Asher L, Collins LM (2019). Positive and negative contexts predict vocal parameters differently in pigs. Anim Behav.

[9] Briefer EF, Tettamanti F, McElligott AG (2015). Emotions in goats: mapping physiological, behavioural and vocal profiles. Anim Behav.

[10] Papadaki K, Bizelis I, Laliotis GP (2021). Acoustic variation in sheep under different social and management conditions. Appl Anim Behav Sci.

[11] Dwyer CM (2008). The welfare of the neonatal lamb. Small Rumin Res.

[12] Searby A, Jouventin P (2003). Mother–lamb acoustic recognition in sheep: a frequency coding. Proc Biol Sci.

[13] Sèbe F, Nowak R, Poindron P, Aubin T (2007). Establishment of vocal communication and discrimination between ewes and their lamb in the first two days after parturition. Dev Psychobiol.

[14] Terrazas A, Serafín N, Hernández H, Nowak R, Poindron P (2012). Early recognition of newborn lambs by ewes. Small Rumin Res.

[15] Papadaki K, Bizelis I, Laliotis GP (2025). Mixing groups of sheep changes vocalizations when an individual animal is isolated. Discover Animals.

[16] Bradley MM, Codispoti M, Cuthbert BN, Lang PJ (2001). Emotion and motivation I: defensive and appetitive reactions in picture processing. Emotion.

[17] Mellor DJ (2015). Positive animal welfare states and encouraging environment-focused and animal-to-animal interactive behaviours. N Z Vet J.

[18] Green AC, Johnston IN, Clark CEF (2019). Sheep recognise familiar and unfamiliar individuals by their bleats. Appl Anim Behav Sci.

[19] Boersma P, Weenink D (2026). Praat: doing phonetics by computer (computer program).

[20] Laliotis GP, Papadaki K, Bizelis I (2023). Ovine vocal individuality expression by ewes and lambs at a late (40 days) post-partum time point. J Acoust Soc Am.

[21] Osiecka AN, Briefer EF, Kidawa D, Wojczulanis-Jakubas K (2024). Strong individual distinctiveness across the vocal repertoire of a colonial seabird, the little auk (Alle alle). Anim Behav.

[22] Reby D, McComb K (2003). Anatomical constraints generate honesty: acoustic cues to age and weight in the roars of red deer stags. Anim Behav.

[23] Hair JF, Black WC, Babin BJ, Anderson RE (2019). Multivariate data analysis.

[24] Celozi S, Miot Z, Renoud-Goud P, Mattiello S, Briefer E, Villain AS (2026). Acoustic markers of negative arousal in lambs: evidence from behavioural and eye thermal profiles. Appl Anim Behav Sci.

[25] Volodin IA, Volodina EV, Sibiryakova OV, Naidenko SV, Hernandez-Blanco JA, Litvinov MN (2015). Vocal activity of the red deer and the acoustic structure of its rutting calls in the Russian far East. Dokl Biol Sci.

[26] Fitch WT (1997). Vocal tract length and formant frequency dispersion correlate with body size in rhesus macaques. J Acoust Soc Am.

[27] Fitch WT, Hauser MD, Simmons AM, Fay RR, Popper AN (2002). Unpacking “honesty”: vertebrate vocal production and the evolution of acoustic signals. Acoustic Communication.

[28] Güzel BC, İşbilir F (2024). Morphometric analysis of the skulls of a ram and ewe Romanov sheep (Ovis aries) with 3D modelling. Vet Med Sci.

[29] Mohamed R, Driscoll M, Mootoo N (2016). Clinical anatomy of the skull of the Barbados Black Belly sheep in Trinidad. Int J Curr Res Med Sci..

[30] Briefer EF, McElligott AG (2012). Social effects on vocal ontogeny in an ungulate, the goat (Capra hircus). Anim Behav.

[31] Frantzola A, Ntairis A, Laliotis GP (2025). Vocal signatures in rams: exploring individual distinctiveness across different contexts. Ruminants.

[32] de la Torre PM, Briefer EF, Reader T, McElligott AG (2015). Acoustic analysis of cattle (Bos taurus) mother–offspring contact calls from a source–filter theory perspective. Appl Anim Behav Sci.

[33] Maigrot AL, Hillmann E, Briefer EF (2018). Encoding of emotional valence in wild boar (Sus scrofa) calls. Animals.

[34] Briefer EF, Sypherd CCR, Linhart P, Leliveld LMC, de la Torre MP, Read ER (2022). Classification of pig calls produced from birth to slaughter according to their emotional valence and context of production. Sci Rep.

[35] Maigrot AL, Hillmann E, Anne C, Briefer EF (2017). Vocal expression of emotional valence in Przewalski’s horses (Equus przewalskii). Sci Rep.

[36] Holeckova S, Policht R (2014). Acoustic divergence in domestic horses. Agric Trop Subtrop.

[37] Charlton BD, Reby D (2011). Context-related acoustic variation in male fallow deer (dama dama) groans. PLoS One.

