# Supplementary material for: Investigating context-specific acoustic variation in the bleats of male domestic sheep
Source: Open Life Sci. 2026 Aug 31;21(1):20251368. doi: 10.1515/biol-2025-1368 (PMC13539161; doi:10.1515/biol-2025-1368)
Supplement: Supplementary file 1 [file j_biol-2025-1368_suppl_001.docx]

Supplementary Table 1: Mean values (± std. error) of each analyzed acoustic parameter in each context (Contexts: A: morning isolation; B: bells hearing; C: night isolation; D: feed anticipation; E: feed denial).en

| <!--Col Count:6-->Acoustic Parameter | Context | | | | |
| --- | --- | --- | --- | --- | --- |
|  | A | B | C | D | E |
| Dur | 0.78 ± 0.02 | 0.80 ± 0.02 | 0.70 ± 0.02 | 0.8 4± 0.02 | 0.88 ± 0.02 |
| Q50 | 695.27 ± 32.55 | 594.66 ± 23.75 | 690.66 ± 22.84 | 557.41 ± 23.31 | 460.10 ± 21.63 |
| Q25 | 396.73 ± 22.87 | 374.73 ± 21.98 | 437.71 ± 16.89 | 262.14 ± 14.96 | 213.62 ± 17.33 |
| Q75 | 1357.66 ± 42.62 | 1164.23 ± 36.24 | 1282.71 ± 40.49 | 1153.36 ± 37.05 | 958.74 ± 35.99 |
| fpeak | 480.19 ± 36.01 | 527.12 ± 26.87 | 536.58 ± 26.25 | 476.58 ± 28.34 | 471.26 ± 31.24 |
| F0TimeMax | 0.41 ± 0.04 | 0.35 ± 0.03 | 0.37 ± 0.03 | 0.39 ± 0.02 | 0.42 ± 0.03 |
| F0mean | 147.60 ± 7.85 | 171.65 ± 9.46 | 157.96 ± 7.24 | 154.62 ± 4.93 | 174.72 ± 8.33 |
| F0AbSlope | 1172.45 ± 74.69 | 1067.58 ± 100.65 | 1133.09 ± 76.71 | 1120.87 ± 63.52 | 1429.98 ± 115.16 |
| F0Range | 203.21 ± 20.47 | 192.61 ± 14.43 | 203.84 ± 16.99 | 229.68 ± 12.85 | 284.41 ± 18.41 |
| F0VAR | 1018.28 ± 69.17 | 948.40 ± 89.23 | 1015.39 ± 70.88 | 990.55 ± 54.89 | 1225.90 ± 97.21 |
| FMextent | 51.78 ± 3.81 | 47.33 ± 5.15 | 48.02 ± 3.43 | 57.24 ± 5.39 | 63.65 ± 5.59 |
| F1mean | 695.03 ± 18.20 | 624.71 ± 9.24 | 645.43 ± 9.06 | 764.90 ± 14.14 | 649.84 ± 12.37 |
| F2mean | 1538.27 ± 17.99 | 1515.03 ± 14.33 | 1525.22 ± 11.83 | 1615.18 ± 17.15 | 1567.34 ± 12.52 |
| F3mean | 2367.12 ± 38.00 | 2278.38 ± 23.14 | 2304.07 ± 21.07 | 2467.91 ± 22.71 | 2403.16 ± 17.71 |
| F4mean | 3456.44 ± 26.64 | 3403.63 ± 17.43 | 3400.23 ± 21.90 | 3547.15 ± 19.76 | 3495.27 ± 14.83 |
| Jitter% | 7.48 ± 0.23 | 6.49 ± 0.24 | 6.94 ± 0.26 | 6.73 ± 0.20 | 6.91 ± 0.24 |
| Shimmer% | 20.94 ± 0.45 | 19.57 ± 0.50 | 21.07 ± 0.45 | 19.41 ± 0.38 | 19.72 ± 0.42 |
| AmpVar | 116.58 ± 5.17 | 101.80 ± 3.49 | 108.72 ± 5.02 | 94.55 ± 2.63 | 98.94 ± 3.73 |
| AMRATE | 18.51 ± 0.47 | 18.58 ± 0.44 | 18.10 ± 0.49 | 19.01 ± 0.30 | 19.82 ± 0.42 |
| VTLmax | 30.30 ± 3.02 | 28.88 ± 2.42 | 26.74 ± 2.13 | 29.15 ± 1.61 | 27.68 ± 1.88 |

Supplementary Table 2: Summary of Canonical Discriminant Functions including: Eigenvalues, Variance Explained (%), Cumulative Variance (%). and Correlationsen

| <!--Col Count:5-->Function | Eigenvalue | % Variance | Cumulative (%) | Correlation |
| --- | --- | --- | --- | --- |
| 1 | 0.85 | 63.6 | 63.6 | 0.68 |
| 2 | 0.39 | 29.3 | 92.9 | 0.53 |
| 3 | 0.06 | 4.9 | 97.8 | 0.25 |
| 4 | 0.03 | 2.2 | 100 | 0.17 |

Supplementary Table 3: Classification Results (%) of the Discriminant Function Analysis, including Original and Cross-Validated Contexts’ Assignmentsen

| <!--Col Count:8-->Method |  | Classification Prediction (%) | | | | | |
| --- | --- | --- | --- | --- | --- | --- | --- |
|  | Context | A | B | C | D | E | Total |
| **Original cases** | **1** | 80.0 | 0 | 20.0 | 0 | 0 | 100 |
|  | **2** | 11.1 | 33.3 | 22.2 | 11.1 | 22.2 | 100 |
|  | **3** | 14.3 | 28.6 | 57.1 | 0 | 0 | 100 |
|  | **4** | 15.4 | 15.4 | 7.7 | 53.8 | 7.7 | 100 |
|  | **5** | 11.1 | 33.3 | 11.1 | 0 | 44.4 | 100 |
| **Cross-validated (leave-one-out)** | **1** | 40.0 | 0 | 20.0 | 20.0 | 20.0 | 100 |
|  | **2** | 11.1 | 33.3 | 22.2 | 11.1 | 22.2 | 100 |
|  | **3** | 42.9 | 42.9 | 14.3 | 0 | 0 | 100 |
|  | **4** | 15.4 | 23.1 | 7.7 | 46.2 | 7.7 | 100 |
|  | **5** | 22.2 | 33.3 | 11.1 | 11.1 | 22.2 | 100 |

Supplementary Table 4: DFA centroid distances between the five experimental contexts (A–E). Distances quantify multivariate acoustic separation, with lower values representing greater acoustic similarity between contexts, whereas larger values reflect stronger multivariate separation of vocal characteristics.en

| <!--Col Count:6-->Context | A | B | C | D | E |
| --- | --- | --- | --- | --- | --- |
| **A** | 0 | 1.529 | 0.627 | 2.045 | 2.134 |
| **B** | 1.529 | 0 | 1.127 | 1.824 | 0.918 |
| **C** | 0.627 | 1.127 | 0 | 2.237 | 1.926 |
| **D** | 2.045 | 1.824 | 2.237 | 0 | 1.337 |
| **E** | 2.134 | 0.918 | 1.926 | 1.337 | 0 |
